# A Multicompartment SIS Stochastic Model with Zonal Ventilation for the Spread of Nosocomial Infections: Detection, Outbreak Management, and Infection Control

**DOI:** 10.1111/risa.13300

**Published:** 2019-03-29

**Authors:** Martín López‐García, Marco‐Felipe King, Catherine J. Noakes

**Affiliations:** ^1^ Department of Applied Mathematics, School of Mathematics University of Leeds Leeds UK; ^2^ Institute for Public Health and Environmental Engineering, School of Civil Engineering University of Leeds Leeds UK

**Keywords:** Airborne spread, nosocomial infections, outbreak detection, SIS stochastic model, summary statistic

## Abstract

In this work, we study the environmental and operational factors that influence airborne transmission of nosocomial infections. We link a deterministic zonal ventilation model for the airborne distribution of infectious material in a hospital ward, with a Markovian multicompartment SIS model for the infection of individuals within this ward, in order to conduct a parametric study on ventilation rates and their effect on the epidemic dynamics. Our stochastic model includes arrival and discharge of patients, as well as the detection of the outbreak by screening events or due to symptoms being shown by infective patients. For each ventilation setting, we measure the infectious potential of a nosocomial outbreak in the hospital ward by means of a summary statistic: the number of infections occurred within the hospital ward until end or declaration of the outbreak. We analytically compute the distribution of this summary statistic, and carry out local and global sensitivity analysis in order to identify the particular characteristics of each ventilation regime with the largest impact on the epidemic spread. Our results show that ward ventilation can have a significant impact on the infection spread, especially under slow detection scenarios or in overoccupied wards, and that decreasing the infection risk for the whole hospital ward might increase the risk in specific areas of the health‐care facility. Moreover, the location of the initial infective individual and the protocol in place for outbreak declaration both form an interplay with ventilation of the ward.

## 1. INTRODUCTION

The risk of acquiring nosocomial infections is a recognized problem in health‐care facilities worldwide (Harbarth, Sax, & Gastmeier, 2003). While the transmission routes for some diseases are well documented, the precise mode of transmission is uncertain for many infections, particularly for those pathogens that cause health‐care–acquired infections (HCAIs). Although it is probable that the majority of transmission occurs via contact routes (Sax et al., 2009), there is increasing recognition that the air plays an important role in disease spread (Li et al., 2007). Understanding the role that ventilation airflow plays in the dispersion of infectious microorganisms is tantamount to assessing exposure to pathogens and hence infection risk. This study aims to provide an analytical link between airborne hospital infection spread, ventilation design, and outbreak management.

Ventilation has been found to have a significant impact on the distribution of infectious material in hospital settings. Examples include *Influenza A* (e.g. H5N1 and H7N9) (Reed & Kemmerly, 2009), *Mycobacterium tuberculosis* (Escombe et al., 2010), measles (Atkinson, Chartier, Pessoa‐Silva, Jensen, & Li, 2009), and *norovirus* (Teunis et al., 2008). One of the most infamous examples occurred in 2003 during the severe acute respiratory syndrome (SARS) outbreak in Hong Kong. Analysis of airflow patterns and outbreak data demonstrated that ventilation routes were critical in the short‐ and long‐range spread of aerosolized *coronavirus* (Li, Huang, Yu, Wong, & Qian, 2005). Ventilation is recognized as an important infection control approach in health‐care design, with strategies such as mechanical ventilation and pressure zoning set out in international (Atkinson et al., 2009) and national guidance (Department of Health, 2007).

Evaluating the influence of ventilation on infection risk typically applies models such as the Wells–Riley equation (Riley, Murphy, & Riley, 1978) or a dose–response approach (Sze & Chao, 2010) to estimate the influence of ventilation on the number of new cases of an infection. Liao, Chang, and Liang (2005) presented a probabilistic transmission dynamic model to assess indoor airborne infection risks and Ko, Burge, Nardell, and Thompson (2001) and Ko, Thompson, and Nardell (2004) developed models for tuberculosis spread incorporating a zonal ventilation model. A number of authors have also looked at control strategies, including Wein and Atkinson (2009), who modeled infection control measures for pandemic influenza; Brienen, Timen, Wallinga, Van Steenbergen, and Teunis (2010), who analyzed the effect of mask use on the spread of influenza; and King, Noakes, and Sleigh (2015), who developed a stochastic model to link airborne and contact transmission. It is also worth mentioning the recent work by Carruthers et al. (2018), where a zonal ventilation model similar to the one considered in this article is linked to a dose–response approach to estimate the risk of infection after an accidental release of bacteria *Francisella tularensis* in a microbiology laboratory.

While these studies enable some understanding of the influence of the environment on transmission, they do not consider relationships between ventilation parameters and the progression and control of an infection outbreak. In an earlier study, it was demonstrated that the Wells–Riley model could be coupled to an SI epidemic model to relate ventilation rate and transmission in a fully mixed environment (Noakes, Beggs, Sleigh, & Kerr, 2006). In later work, a zonal air distribution and a stochastic formulation (Noakes & Sleigh, 2009) was considered, and cost benefits of ventilation from an energy and infection risk perspective were explored (Noakes, Sleigh, & Khan, 2012).

The model presented in this article is constructed on a scenario defined in previous work (Noakes & Sleigh, 2009), where the role played by the airflow during a nosocomial outbreak is assessed by linking a deterministic zonal ventilation model with an SI stochastic epidemic model using a computational approach. While the previous approach enabled exploration of the basic interaction between the ventilation and the outbreak, there are a number of limitations:

- The epidemic dynamics are represented through a simple SI epidemic model, not accounting for relevant factors such as the discharge and admission of patients, or the detection and declaration of the outbreak.
- Results reported by Noakes and Sleigh (2009) have high variability, which is related to the fact that they were obtained by means of stochastic simulations of the epidemic process.
- The large number of parameters associated with each ventilation scenario makes it difficult to identify, from stochastic simulations, the specific factors of the ventilation air distribution that facilitate or mitigate epidemic spread.

We refer the reader to Keeling and Ross (2007) where the limitations of analyzing this type of epidemic processes by simulation are discussed in more detail, and where the benefits of following exact analytical approaches instead are highlighted.

Our aim here is to show how this zonal ventilation model can be linked to more complex stochastic epidemic models for the spread of nosocomial pathogens, while accounting for patients, admission and discharge, and different outbreak detection and declaration hypotheses. We show how to implement exact analytical procedures for computing summary statistics of the outbreak (statistics measuring outbreak infectiousness), which by means of a perturbation analysis enables identification of specific characteristics of the ventilation setting that are crucial for the spread or control of the infection.

Finally, we carry out a comprehensive numerical study of six ventilation strategies for a hypothetical hospital ward in order to identify particular ventilation characteristics that may promote or inhibit spread of airborne nosocomial infections. Our results explore the interplay between ward ventilation, location of patients, ward overoccupancy, and outbreak detection management.

## 2. THE MODEL

Noakes and Sleigh (2009) propose a stochastic model that links a zonal ventilation model with epidemic dynamics by means of an *SI* model for the infection spread among individuals in a hospital setting. The combined model can be thought of as an adaptation of the Wells–Riley model (Riley et al., 1978; Sze & Chao, 2010), where each susceptible individual is infected with a per capita rate,

$$\begin{array}{ccc} \lambda & = & \frac{Ipq}{Q}, \end{array}$$

that is proportional to the number *I* of infective individuals. Here, *Q* is the room ventilation rate (m^3^/s), *p* is the pulmonary ventilation (breathing) rate (m^3^/s), and *q* is the unit of infection (*quantum*) as described by Riley et al. (1978). In a standard SI stochastic epidemic model, where individuals are either susceptible or infective, individuals do not recover. This can be expressed in terms of a continuous‐time Markov chain (CTMC) {S(t):t≥0}, where the random variable S(t) represents the number of susceptible individuals at time *t*, and the aim is to compute transient probabilities $p_{S}\left(t\right)=P\left(S\left(t\right)=S\right)$ for any S∈Ω={0,1,⋯,N}, where *N* is the number of individuals in the population. These probabilities satisfy the Kolmogorov differential equations:

$$\begin{array}{ccc} \frac{dp_{S}\left(t\right)}{dt} & = & -\lambda Sp_{S}\left(t\right)+\lambda \left(S+1\right)p_{S+1}\left(t\right),\;S\in \Omega . \end{array}$$

Interevent times are assumed to be exponentially distributed and the stochastic process is simulated using the Gillespie algorithm (Gillespie, 1976). In particular, this algorithm generates samples of the interevent times by using the fact that

(1) $$\begin{array}{ccc} U∼U(0,1) & \Rightarrow & T=-\frac{\ln(U)}{\lambda S}∼\exp\left(\lambda S\right), \end{array}$$

and then updates the state of the system according to the probabilities of each possible event occurring.

### 2.1. A Zonal Ventilation Model for Linking Airflow Dynamics and Infection Rates

In Noakes and Sleigh (2009), this Wells–Riley process is adapted to investigate ventilation scenarios in a hypothetical hospital ward split in *M* *ventilation zones*. The air is assumed to be uniformly mixed within each zone; however, there is incomplete mixing between the zones and unbalanced zone boundaries allow for the effect of directional flow to be examined. In particular, the per capita infection rate $\lambda_{k}$ for susceptible individuals at zone *k* is defined as $\lambda_{k}=p_{k}C_{k}$, where $C_{k}$ is the concentration of infectious material at zone *k* and $p_{k}$ is the pulmonary rate of these individuals. We note that this concentration could depend on the number of infected individuals, $i_{j}$, in every zone 1≤j≤M, due to airflow. In Noakes and Sleigh (2009), the spatial distribution of infectious material is represented through the differential equation:

(2) $$\begin{array}{ccc} V_{k}\frac{dC_{k}}{dt} & = & q_{k}i_{k}-Q_{o,k}C_{k}-\sum_{j}\beta_{kj}C_{k}+\sum_{j}\beta_{jk}C_{j},\\ & & 1\leq k\leq M, \end{array}$$

where $V_{k}$ is the volume of zone *k*, $q_{k}i_{k}$ is the generation rate of infectious quanta, $Q_{o,k}$ represents the extract ventilation rate in zone *k*, and $\sum_{j}\beta_{kj}C_{k}$ and $\sum_{j}\beta_{jk}C_{j}$ amount to the volume flow of air between zones *k* and *j*. Moreover, each interzonal flow rate $\beta_{kj}$ represents the sum of two contributions:

$$\beta_{kj}=\beta_{0}+\beta_{Qkj},$$

where β_0_ is a global mixing rate and $\beta_{Qkj}$ is an additional contribution representing net flow across the k/j zonal boundary, from zone *k* to zone *j*.

Equation (2) leads to a *ventilation matrix* that characterizes the ventilation air distribution under study,

$$\begin{array}{ccc} V & = & \left(\begin{array}{ccccc} Q_{o,1}+\sum_{k}\beta_{1k} & -\beta_{21} & \cdots & -\beta_{M-1,1} & -\beta_{M1}\\ -\beta_{12} & Q_{o,2}+\sum_{k}\beta_{2k} & \cdots & -\beta_{M-1,2} & -\beta_{M2}\\ -\beta_{13} & -\beta_{23} & \cdots & -\beta_{M-1,3} & -\beta_{M3}\\ \vdots & \vdots & \ddots & \vdots & \vdots \\ -\beta_{1M} & -\beta_{2M} & \cdots & -\beta_{M-1,M} & Q_{o,M}+\sum_{k}\beta_{Mk} \end{array}\right) \end{array}$$

representing ventilation in a hospital ward divided into *M* ventilation zones.

By assuming steady‐state conditions for airflow, and taking into account Equation (2), Noakes and Sleigh (2009) propose to link infection rates $\lambda_{j}$, j∈{1,⋯,M} with the ventilation matrix **V** as follows:

(3) $$\begin{array}{ccc} \left(\begin{array}{c} \frac{\lambda_{1}}{p_{1}}\\ \frac{\lambda_{2}}{p_{2}}\\ \vdots \\ \frac{\lambda_{M}}{p_{M}} \end{array}\right) & = & V^{-1}\left(\begin{array}{c} q_{1}i_{1}\\ q_{2}i_{2}\\ \vdots \\ q_{M}i_{M} \end{array}\right). \end{array}$$

This means that per capita infection rates $\left\{\lambda_{1},\cdots ,\lambda_{M}\right\}$ for susceptible individuals at zones {1,⋯,M} depend on how many infective individuals $\left(i_{1},\cdots ,i_{M}\right)$ there are in any zone at any given time, computed in a specialized manner (Equation (3)) that takes into account the ventilation distribution through matrix **V**. Once this procedure for computing infection rates is proposed, stochastic simulations for the SI epidemic dynamics are carried out in Noakes and Sleigh (2009) by following steps (i)–(iv) (Noakes & Sleigh, 2009), which assume exponentially distributed interevent times and make use of the property depicted in Equation (1).

We note here that Equation (3) means considering per capita infection rates at each zone 1≤j≤M as functions $\lambda_{j}\left(i_{1},\cdots ,i_{M}\right)$ of the number of infectives $\left(i_{1},\cdots ,i_{M}\right)$ within each ventilation zone at the hospital ward. In Section 2.2, we now go on to exploit this, to link the zonal ventilation model with a multicompartment SIS model with detection, to evaluate the infection spread dynamics within the hospital ward until detection of the outbreak. Instead of carrying out stochastic simulations, we present an exact approach for analyzing a summary statistic of the outbreak: the total number of infections occurring until the outbreak ends or is detected and declared. This exact approach does not only allow us to compute this quantity of interest, but it also allows one to carry out a sensitivity analysis on the model parameters, so that the impact that different characteristics of the ventilation setting has on this summary statistic can be evaluated.

### 2.2. A Multicompartment SIS Stochastic Model for the Infection Spread Dynamics

**Figure 1:**
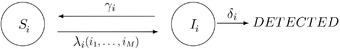
Individual epidemic dynamics for a patient in zone *i*. Event $I_{i}\to S_{i}$ represents the discharge of the infective patient in zone *i*, immediately replaced by a susceptible one.

At the epidemic level, we assume that patients in each zone *i* are discharged at rate $\gamma_{i}$, so that $\gamma_{i}^{-1}$ amounts to the average length of stay (LOS) of patients in zone *i*. Discharges are immediately replaced by new admitted patients, a reasonable approximation for hospital wards under high demand (Pelupessy, Bonten, & Diekmann, 2002; Wolkewitz, Dettenkofer, Bertz, Schumacher, & Huebner, 2008). Moreover, we consider that the nosocomial outbreak will go undetected by health‐care workers for some time, and incorporate this fact into our model by considering that each infected individual in zone *i* can be *discovered*/*detected* at some rate $\delta_{i}$. The reciprocal $\delta_{i}^{-1}$ represents the average time until some symptoms arise that alert health‐care workers to a patient's infection, or the average time until the infected individual is detected through screening policies put in place at this hospital ward. Fig. 1 represents the epidemic dynamics for an individual in zone *i*.

This leads to a multicompartment SIS epidemic model that can be described as a CTMC $X=\{\left(I_{1}\left(t\right),\cdots ,I_{M}\left(t\right)\right):\;t\geq 0\}$, where $I_{j}\left(t\right)$ represents the number of infective individuals in zone *j* at time t≥0, defined over the space of states $S=\left\{\left(i_{1},i_{2},\cdots ,i_{M}\right):\;i_{j}\in \left\{0,\cdots ,N_{j}\right\},\;j\in \left\{1,\cdots ,M\right\}\right\}\cup \left\{\Delta \right\}=C\cup \left\{\Delta \right\}$. $N_{j}$ is the total number of patients in zone *j*, leading to $N=\sum_{j=1}^{M}N_{j}$ patients in the hospital ward. State Δ represents that the nosocomial outbreak has been detected and declared by health‐care workers by the first detection of an infected patient in the hospital ward. We note that absorbing state (0, …, 0) represents the end of the outbreak (lack of infective individuals), due to patients' discharge (i.e., if all the patients infected by the pathogen are discharged before the outbreak is actually detected). We consider Δ also as an absorbing state in this process, since we are only interested in the dynamics of the process until the end or declaration of the outbreak, and the transitions (obtained from diagram in Fig. 1) described in Table I. We note that, according to our comments in Section 2.1, $\lambda_{j}\left(i_{1},\cdots ,i_{M}\right)$ is a function of the state $\left(i_{1},\cdots ,i_{M}\right)$, representing the per capita infection rate of susceptible individuals in zone *j* when we have $\left(i_{1},\cdots ,i_{M}\right)$ infective individuals within the ward, computed from Equation (3) for each $(i_{1},\cdots ,i_{M})\in C$.

**Table I:** Transitions or Events Occurring in the Stochastic Process X, and Corresponding Rates

Event	Stochastic Transition	Rate
Infection of a patient in zone *j*	$\left(i_{1},\cdots ,i_{j},\cdots ,i_{M}\right)\to \left(i_{1},\cdots ,i_{j}+1,\cdots ,i_{M}\right)$	$\lambda_{j}\left(i_{1},\cdots ,i_{M}\right)\left(N_{j}-i_{j}\right)$
Discharge of an infective patient in zone *j*	$\left(i_{1},\cdots ,i_{j},\cdots ,i_{M}\right)\to \left(i_{1},\cdots ,i_{j}-1,\cdots ,i_{M}\right)$	$\gamma_{j}i_{j}$
Detection of the outbreak	$(i_{1},\cdots ,i_{M})\to \Delta$	$\sum_{j=1}^{M}\delta_{j}i_{j}$

## 3. METHODOLOGY

Our interest is in analyzing the infectious potential of an outbreak in a given hospital ward, for different ventilation configurations and outbreak detection hypotheses. We measure the infectious potential in terms of the following discrete random variable:

$$\begin{array}{ccc} R & = & \text{number}\;\text{of}\;\text{infections}\;\text{occurred}\;\text{until}\;\text{the}\;\text{end}\;\text{or}\\ & & \text{declaration}\;\text{of}\;\text{the}\;\text{outbreak,} \end{array}$$

which can take values in {0,1,2,⋯}. *R* is used here as a measure of how well ventilation can act as a *preventive infection control strategy* that is in place even before the actual detection of the outbreak by health‐care workers occurs and *reactive* strategies can be implemented.

### 3.1. Analyzing *R*

For an initial state $(i_{1},\cdots ,i_{M})\in C$, our aim is to compute probabilities

$$\begin{array}{ccc} p_{\left(i_{1},i_{2},\cdots ,i_{M}\right)}\left(n\right) & = & P(R=n\;|\;\left(I_{1}\left(0\right),I_{2}\left(0\right),\cdots ,I_{M}\left(0\right)\right)\\ & = & (i_{1},i_{2},\cdots ,i_{M})), \end{array}$$

for n≥0; that is, the probability distribution of *R* for some initial state $\left(i_{1},\cdots ,i_{M}\right)$. We can compute these probabilities from a system of linear equations, which is obtained by a first‐step argument. In particular, by proposing notation

$$\begin{array}{ccc} i & = & (i_{1},\cdots ,i_{M}),\\ i^{+}\left(s\right) & = & (i_{1},\cdots ,i_{s}+1,\cdots ,i_{M}),\\ i^{-}\left(s\right) & = & (i_{1},\cdots ,i_{s}-1,\cdots ,i_{M}), \end{array}$$

we get

(4) $$\begin{array}{ccc} & & p_{i}\left(0\right)\sum_{j=1}^{M}\left(\lambda_{j}\left(i\right)\left(N_{j}-i_{j}\right)+\left(\gamma_{j}+\delta_{j}\right)i_{j}\right)\\ & & \;\;\;=\;\sum_{k=1}^{M}\left(\gamma_{k}i_{k}p_{i^{-}\left(k\right)}\left(0\right)+\delta_{k}i_{k}\right), \end{array}$$

(5) $$\begin{array}{ccc} & & p_{i}\left(n\right)\sum_{j=1}^{M}\left(\lambda_{j}\left(i\right)\left(N_{j}-i_{j}\right)+\left(\gamma_{j}+\delta_{j}\right)i_{j}\right)\\ & & =\;\sum_{k=1}^{M}\left(\gamma_{k}i_{k}p_{i^{-}\left(k\right)}\left(n\right)+\lambda_{k}\left(i\right)\left(N_{k}-i_{k}\right)p_{i^{+}\left(k\right)}\left(n-1\right)\right), \end{array}$$

for n≥1 and any $(i_{1},\cdots ,i_{M})\in C$, and with boundary conditions $p_{\left(0,0,\cdots ,0\right)}\left(0\right)=1$, $p_{\left(0,0,\cdots ,0\right)}\left(n\right)=0$ for all n≥1; a detailed explanation on how Equations (4) and (5) are obtained is in the Appendix. This means that probabilities for n=0 (Equation (4)) can be computed by solving a system of

(6) $$\begin{array}{ccc} \#C & = & \prod_{i=1}^{M}\left(N_{i}+1\right) \end{array}$$

linear equations.^1^ Once these are in hand, probabilities for n≥1 can be computed by solving the system of linear equations given by Equation (5), which also consists of #C equations.

Algorithm 1 in the Appendix computes probabilities $p_{\left(i_{1},\cdots ,i_{M}\right)}\left(n\right)$ for any $(i_{1},\cdots ,i_{M})\in C$, n≥0. It works sequentially, computing probabilities $p_{\left(i_{1},\cdots ,i_{M}\right)}\left(n\right)$ for $i_{1}+i_{2}+\cdots +i_{M}=I$ for increasing values of n=0,1,2,⋯ and I=1,2,3,⋯,N.

### 3.2. Local Sensitivity Analysis

Our analysis allows one to identify the most important characteristics of the ventilation scenario, regarding the infectious potential of the outbreak until detection, by means of computing partial derivatives of the form ∂E[R]/∂θ with respect to ventilation parameters $\theta \in \left\{\beta_{0}\right\}\cup \left\{\beta_{Qij}:\;i,j\in \left\{1,\cdots ,M\right\}\right\}\cup \left\{Q_{o,i}:\;i\in \left\{1,\cdots ,M\right\}\right\}$. We note that, for an initial state $(i_{1},\cdots ,i_{M})\in C$, $E\left[R\right]=\sum_{n=0}^{+\infty }np_{\left(i_{1},\cdots ,i_{M}\right)}\left(n\right)$, so that

(7) $$\begin{array}{c} \frac{\partial E[R]}{\partial \theta }=\sum_{n=0}^{+\infty }n\frac{\partial p_{\left(i_{1},\cdots ,i_{M}\right)}\left(n\right)}{\partial \theta }. \end{array}$$

Partial derivatives $\frac{\partial p_{\left(i_{1},\cdots ,i_{M}\right)}\left(n\right)}{\partial \theta }$ can be computed from direct differentiation of Equations (4) and (5). In Equations (4) and (5), the only quantities that depend on parameter $\theta \in \left\{\beta_{0}\right\}\cup \left\{\beta_{Qij}:\;i,j\in \left\{1,\cdots ,M\right\}\right\}\cup \left\{Q_{o,i}:\;i\in \left\{1,\cdots ,M\right\}\right\}$ are infection rates $\lambda_{j}\left(i_{1},\cdots ,i_{M}\right)$ and probabilities $p_{\left(i_{1},\cdots ,i_{M}\right)}\left(n\right)$. Thus, we get

$$\begin{array}{ccc} & & \sum_{j=1}^{M}\frac{\partial \lambda_{j}\left(i\right)}{\partial \theta }\left(N_{j}-i_{j}\right)p_{i}\left(0\right)\;+\;\sum_{j=1}^{M}\left(\lambda_{j}\left(i\right)\left(N_{j}-i_{j}\right)\right.\\ & & \;\left.+\;\left(\gamma_{j}+\delta_{j}\right)i_{j}\right)\frac{\partial p_{i}\left(0\right)}{\partial \theta }\;=\;\sum_{k=1}^{M}\gamma_{k}i_{k}\frac{\partial p_{i^{-}\left(k\right)}\left(0\right)}{\partial \theta },\\ & & \;\;\sum_{j=1}^{M}\frac{\partial \lambda_{j}\left(i\right)}{\partial \theta }\left(N_{j}-i_{j}\right)p_{i}\left(n\right)+\sum_{j=1}^{M}\left(\lambda_{j}\left(i\right)\left(N_{j}-i_{j}\right)\right.\\ & & \;\left.+\;\left(\gamma_{j}+\delta_{j}\right)i_{j}\right)\frac{\partial p_{i}\left(n\right)}{\partial \theta }\;=\;\sum_{k=1}^{M}\left(\gamma_{k}i_{k}\frac{\partial p_{i^{-}\left(k\right)}\left(n\right)}{\partial \theta }\right.\\ & & \;\left.+\;\frac{\partial \lambda_{k}\left(i\right)}{\partial \theta }\left(N_{k}-i_{k}\right)p_{i^{+}\left(k\right)}\left(n-1\right)\right.\\ & & \;\;\left.+\;\lambda_{k}\left(i\right)\left(N_{k}-i_{k}\right)\frac{\partial p_{i^{+}\left(k\right)}\left(n-1\right)}{\partial \theta }\right), \end{array}$$

for n≥1, and any $(i_{1},\cdots ,i_{M})\in C$. Partial derivatives $\frac{\partial p_{\left(i_{1},\cdots ,i_{M}\right)}\left(n\right)}{\partial \theta }$ can then be computed from the equations above by following arguments similar to those in Algorithm 1 in the Appendix. In order to solve these equations, one needs to have in hand values of $p_{\left(i_{1},\cdots ,i_{M}\right)}\left(n\right)$ (previously computed from Algorithm 1), as well as derivatives $\frac{\partial \lambda_{j}\left(i_{1},\cdots ,i_{M}\right)}{\partial \theta }$. These derivatives can be straightforwardly obtained from Equation (3) as:

$$\begin{array}{ccc} \left(\begin{array}{c} \frac{1}{p_{1}}\cdot \frac{\partial \lambda_{1}\left(i_{1},\cdots ,i_{M}\right)}{\partial \theta }\\ \frac{1}{p_{2}}\cdot \frac{\partial \lambda_{2}\left(i_{1},\cdots ,i_{M}\right)}{\partial \theta }\\ \vdots \\ \frac{1}{p_{M}}\cdot \frac{\partial \lambda_{M}\left(i_{1},\cdots ,i_{M}\right)}{\partial \theta } \end{array}\right) & = & -V^{-1}V^{\left(\theta \right)}V^{-1}\left(\begin{array}{c} q_{1}i_{1}\\ q_{2}i_{2}\\ \vdots \\ q_{M}i_{M} \end{array}\right), \end{array}$$

where $V^{\left(\theta \right)}$ represents the element‐by‐element partial derivative of matrix **V** with respect to parameter θ (Gómez‐Corral & López‐García, 2018).

### 3.3. Spread until the *D*th Individual Detection

As outlined above, declaration of the outbreak is identified with the first detection of an infective patient, where each patient is detected in zone *j* at rate $\delta_{j}$. If detection of an infective patient occurs because this patient shows symptoms, outbreak declaration might require several (D>1) patients showing some common symptoms, since for some nosocomial pathogens, associated symptoms are quite common and pass unnoticed (Ekkert, 2015). For example, *norovirus* causes gastrointestinal symptoms such as nausea, vomiting, or diarrhea that are common to many diseases and conditions. The *National Guidelines on the Management of Outbreaks of Norovirus Infection in Health‐Care Settings* (National Disease Surveillance Centre, 2003), issued by the National Disease Surveillance Centre in Ireland, requires for D=2 patients to show these symptoms in a hospital ward for a potential norovirus outbreak declaration. Once the outbreak has been declared, control strategies such as immediate cleaning and decontamination, frequent handwashing, or cohorting of affected patients are recommended.

Thus, our interest in this subsection is to analyze the summary statistic *R* when the detection of the outbreak requires *D* patients to show symptoms, for some value D≥1, and results in the subsections above can be seen as the particular case D=1. We define the augmented process $X^{aug}=\left\{\left(I_{1}\left(t\right),\cdots ,I_{M}\left(t\right),D\left(t\right)\right):\;t\geq 0\right\}$, where the increasing variable D(t) amounts to the number of detected patients up to time t≥0. We consider that the outbreak is declared once D(t)=D, and the space of states of this CTMC is given by:

$$\begin{array}{ccc} S^{aug} & = & \{\left(i_{1},i_{2},\cdots ,i_{M},d\right):\;i_{j}\in \left\{0,\cdots ,N_{j}\right\},\\ & & j\in \{1,\cdots ,M\},\;0\leq d\leq D-1\}\cup \{\Delta \}. \end{array}$$

Thus, $S^{aug}=C^{aug}\cup \Delta$, with state Δ representing outbreak declaration (i.e., the detection of the *D*th infected patient). Events occurring in this process, at different rates, are described in Table II.

**Table II:** Transitions or Events Occurring in the Stochastic Process $X^{aug}$, and Corresponding Rates

Event	Stochastic Transition	Rate
Infection of a patient in zone *j*	$\left(i_{1},\cdots ,i_{j},\cdots ,i_{M},d\right)\to \left(i_{1},\cdots ,i_{j}+1,\cdots ,i_{M},d\right)$	$\lambda_{j}\left(i_{1},\cdots ,i_{M}\right)\left(N_{j}-i_{j}\right)$
Discharge of an infective patient in zone *j*	$\left(i_{1},\cdots ,i_{j},\cdots ,i_{M},d\right)\to \left(i_{1},\cdots ,i_{j}-1,\cdots ,i_{M},d\right)$	$\gamma_{j}i_{j}$
Detection of an infective patient	$\left(i_{1},\cdots ,i_{M},d\right)\to \left(i_{1},\cdots ,i_{M},d+1\right)$, 0≤d≤D−2	$\sum_{j=1}^{M}\delta_{j}i_{j}$
Outbreak declaration	$(i_{1},\cdots ,i_{M},D-1)\to \Delta$	$\sum_{j=1}^{M}\delta_{j}i_{j}$

Our arguments in the subsections above can be adapted for process $X^{aug}$. For example, Equations (4) and (5) become

$$\begin{array}{ccc} & & \;p_{\left(i_{1},\cdots ,i_{M},d\right)}\left(0\right)\sum_{j=1}^{M}\left(\lambda_{j}\left(i_{1},\cdots ,i_{M}\right)\left(N_{j}\;-\;i_{j}\right)\;+\;\left(\gamma_{j}\;+\;\delta_{j}\right)i_{j}\right)\\ & & \;=\;\sum_{k=1}^{M}\left(\gamma_{k}i_{k}p_{\left(i_{1},\cdots ,i_{k}-1,\cdots ,i_{M},d\right)}\left(0\right)\right.\\ & & \;\left.+\;\delta_{k}i_{k}\left(1_{d=D-1}+1_{d<D-1}p_{\left(i_{1},\cdots ,i_{M},d+1\right)}\left(0\right)\right)\right),\\ & & \;p_{\left(i_{1},\cdots ,i_{M},d\right)}\left(n\right)\sum_{j=1}^{M}\left(\lambda_{j}\left(i_{1},\cdots ,i_{M}\right)\left(N_{j}\;-\;i_{j}\right)\;+\;\left(\gamma_{j}\;+\;\delta_{j}\right)i_{j}\right)\\ & & \;=\;\sum_{k=1}^{M}\left(\gamma_{k}i_{k}p_{\left(i_{1},\cdots ,i_{k}-1,\cdots ,i_{M},d\right)}\left(n\right)\right.\\ & & \;\left.+\;\lambda_{k}\left(i_{1},\cdots ,i_{M}\right)\left(N_{k}-i_{k}\right)p_{\left(i_{1},\cdots ,i_{k}+1,\cdots ,i_{M},d\right)}\left(n-1\right)\right.\\ & & \;\left.+\;\delta_{k}i_{k}1_{d<D-1}p_{\left(i_{1},\cdots ,i_{M},d+1\right)}\left(n\right)\right),\;n\geq 1 \end{array}$$

for any $\left(i_{1},\cdots ,i_{M},d\right)\in C^{aug}$, and with boundary conditions $p_{\left(0,0,\cdots ,0,d\right)}\left(0\right)=p_{\Delta }\left(0\right)=1$, and $p_{\left(0,0,\cdots ,0,d\right)}\left(n\right)=p_{\Delta }\left(n\right)=0$ for all n≥1 and 0≤d≤D−1. 1_*A*_ represents a function that takes value 1 if *A* is satisfied, and 0 otherwise. An adapted version of Algorithm 1, not reported here for the sake of brevity, allows for an efficient solution of this system.

## 4. RESULTS

**Figure 2:**
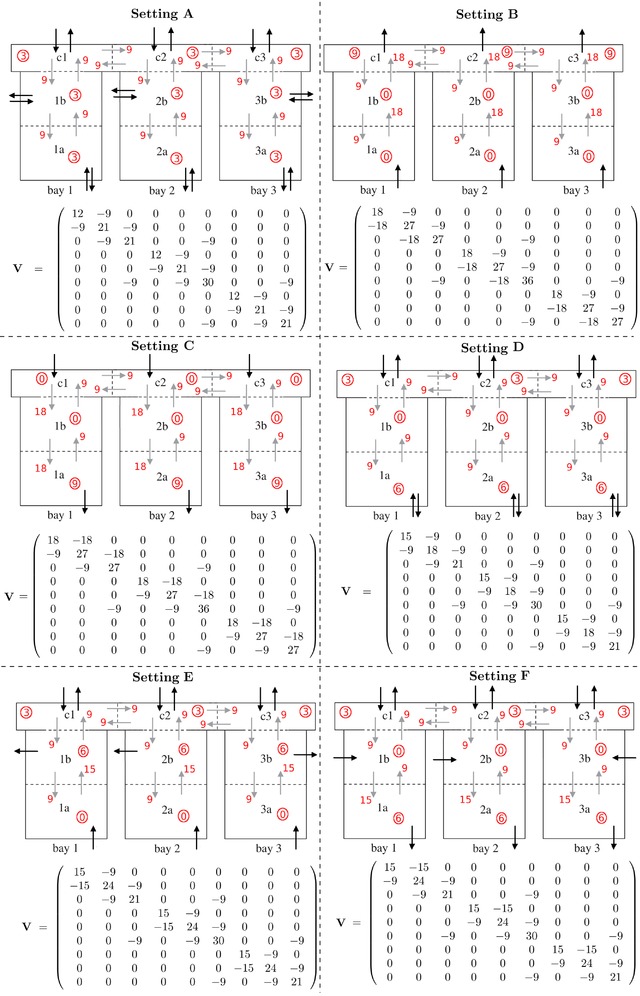
Diagrams of the ventilation settings considered, and corresponding ventilation matrices **V** (for $\beta_{0}=9\;m^{3}/\min$). Gray arrows: ventilation between zones ($\beta_{ij}\;$, m^3^/min); black arrows: ventilation supply and extract to the ward; circled values: extract ventilation rates ($Q_{o,j}$, m^3^/min)

We consider here the hypothetical hospital ward in Fig. 2 in Noakes and Sleigh (2009), and ventilation settings according to Table 1 in Noakes and Sleigh (2009). This hypothetical hospital ward consists of three six‐bedded bays connected through a corridor. Each bay is split into two ventilation zones, and each ventilation zone contains three patients. The corridor is split into three ventilation zones and in the first instance has no patients, so that the ward has N=18 patients. Ventilation rate over the whole ward was $27\;m^{3}/\min$, which equated to an air change rate of 3 AC/h. Diagrams of the ventilation flows and specific rates within and between each zone for each setting are given in Fig. 2. Moreover, we set $p_{i}=0.01\;m^{3}/\min$ and qi=0.5 quanta / min  for all patients at all zones and for every ventilation scenario (Noakes & Sleigh, 2009), and assume an average LOS for each patient $\gamma_{i}^{-1}=7$ days.

If we order the M=9 ventilation zones as

1a≺1b≺c1≺2a≺2b≺c2≺3a≺3b≺c3,

the ventilation matrix is given by

$$\begin{array}{ccc} V & = & \left(\begin{array}{ccccc} Q_{o,1a}+\sum_{k}\beta_{1a,k} & -\beta_{1b,1a} & \cdots & -\beta_{3b,1a} & -\beta_{c3,1a}\\ -\beta_{1a,1b} & Q_{o,1b}+\sum_{k}\beta_{1b,k} & \cdots & -\beta_{3b,1b} & -\beta_{c3,1b}\\ -\beta_{1a,c1} & -\beta_{1b,c1} & \cdots & -\beta_{3b,c1} & -\beta_{c3,c1}\\ \vdots & \vdots & \ddots & \vdots & \vdots \\ -\beta_{1a,c3} & -\beta_{1b,c3} & \cdots & -\beta_{3b,c3} & Q_{o,c3}+\sum_{k}\beta_{c3,k} \end{array}\right) \end{array}$$

and ventilation settings in Table 1 in Noakes and Sleigh (2009) lead to the ventilation matrices reported in Fig. 2.

In Sections 4.1–4.3 and 4.6, we consider that outbreak declaration occurs after one patient shows symptoms, with each patient showing symptoms after an average time $\delta^{-1}$ (i.e., $\delta_{j}=\delta$ for all 1≤j≤M). Alternative outbreak detection and declaration hypotheses are discussed in Sections 4.4 and 4.5, while the impact of parameter *q* in our numerical results is explored in Section 4.7.

### 4.1. Impact of Ventilation Setting on Spread Dynamics

**Figure 3:**
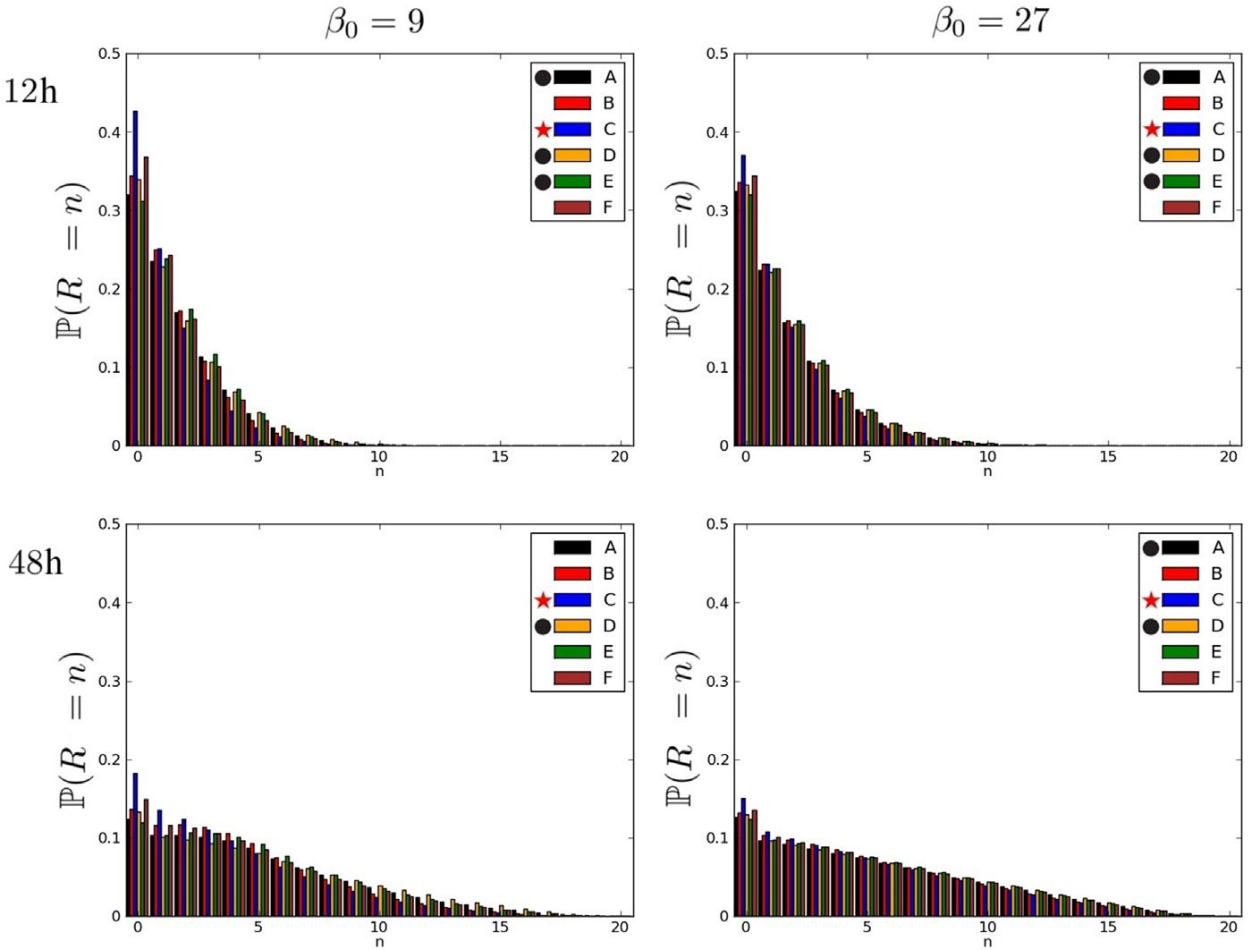
Probability mass function of the number *R* of infections until the end or detection of the outbreak, for ventilation settings A–F. Declaration of the outbreak occurs once one patient shows symptoms, and each infective patient shows symptoms after an average time $\delta^{-1}\in \left\{12\;h,48\;h\right\}$. Global mixing rate $\beta_{0}\in \left\{9,27\right\}\;m^{3}/\min$. Initial infective located in zone 1*a*. Red stars identify the best ventilation settings in terms of *E*[*R*], while black circles identify the worst ones; see Table III.

In Fig. 3, we plot the probability mass function of *R* versus different values of the global mixing rate β_0_, the average time $\delta^{-1}$ at which each infective patient shows symptoms, and for ventilation scenarios A–F. For these results, it is assumed that an infective patient in zone 1*a* starts the outbreak, and we report in Table III the mean values *E*[*R*] computed for these distributions.

**Table III:** Mean Number *E*[*R*] of Infections until End or Declaration of the Outbreak, for Scenarios in Fig. 3

β_0_	$\delta^{-1}$	A	B	C	D	E	F
9	12 h	1.75	1.54	1.24	1.75	1.74	1.50
	48 h	5.01	4.34	3.87	5.20	4.86	4.50
27	12 h	1.88	1.76	1.61	1.86	1.87	1.77
	48 h	5.59	5.25	5.03	5.59	5.53	5.37

Ventilation setting C can be identified in Fig. 3 and Table III as the best one, while settings A, D, and E are identified as the worst ones depending on the detection parameter δ and the global mixing rate β_0_. We note that ventilation setting C has significant extract ventilation at the initially *infected zone* 1*a*, so that the airflow is directed from 1*a* outward to the hospital ward. On the other hand, ventilation setting D represents a well‐mixed ward ($\beta_{Qik}=0$ for all *i* and *k*) with no extract ventilation at zone 1*b* ($Q_{o,1b}=0$), which might favor the spread of pathogens from 1*a* toward other zones within the ward, leading to more infections occurring until outbreak detection.

In general terms, worse scenarios can be identified for values $\delta^{-1}=48$ h and $\beta_{0}=27\;m^{3}/\min$, where the long‐tailed distribution of *R* for these scenarios in Fig. 3 indicates that large outbreaks occur with significant probability. Larger differences among ventilation settings are also found for value $\delta^{-1}=48$ h. Thus, our results suggest that ventilation of the ward should be of special concern for pathogens that have longer infectious asymptomatic periods, or in hospital wards with more limited surveillance policies. It is also clear that the average individual detection time $\delta^{-1}$ has a higher impact on the infection spread than the specific ventilation setting in the ward, so that outbreak detection seems to *dominate* ventilation regarding infection spread.

### 4.2. Dependence on Location of Initial Infective

**Figure 4:**
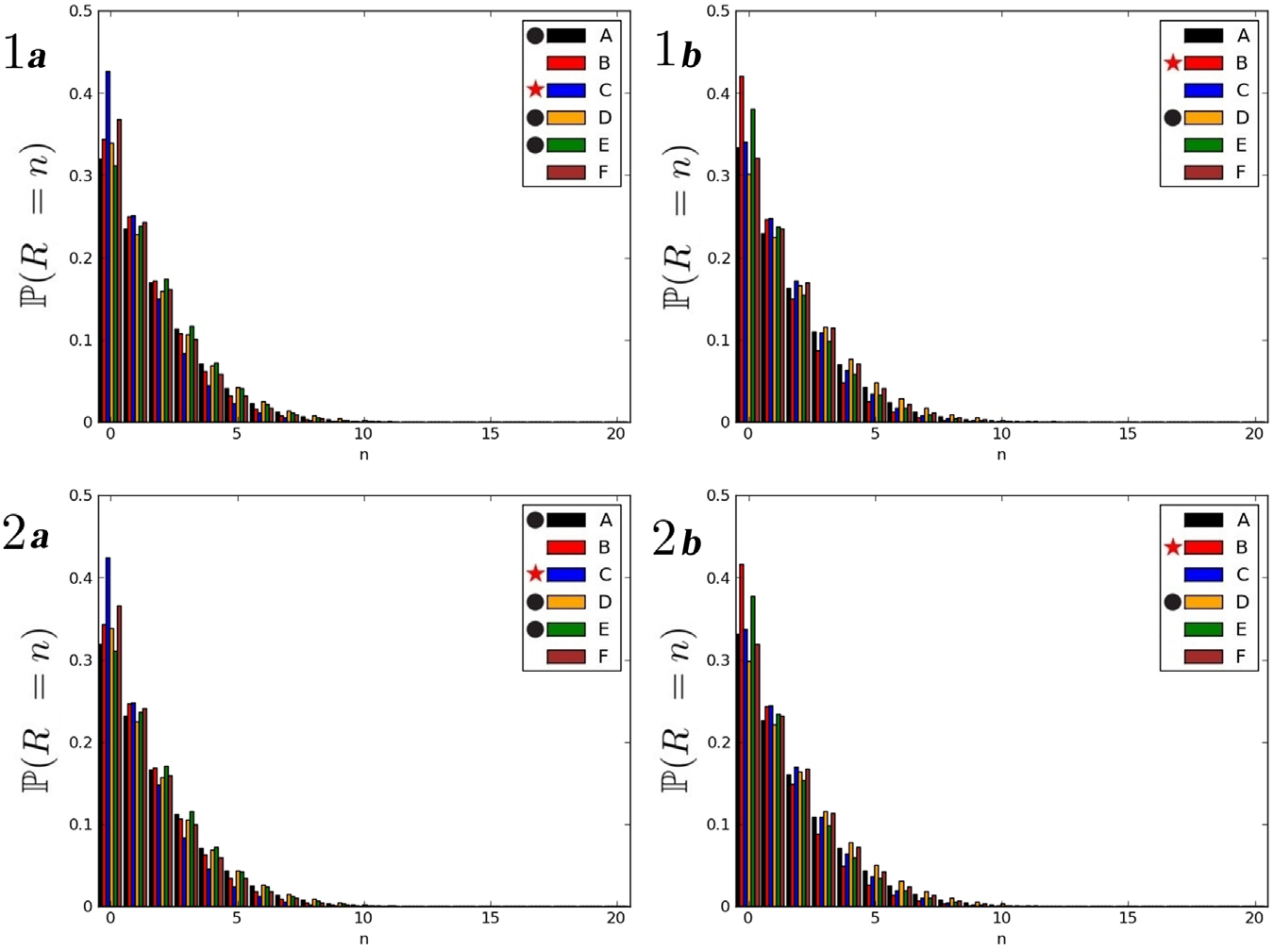
Probability mass function of the number *R* of infections until the end or detection of the outbreak, for ventilation settings A–F. Declaration of the outbreak occurs once one patient shows symptoms, and each infective patient shows symptoms after an average time $\delta^{-1}=12$ h. Initial infective located in zones {1a,1b,2a,2b}. Global mixing rate $\beta_{0}=9\;m^{3}/\min.$

In Fig. 4, we plot analogous results to those in Fig. 3 when the infective patient starting the outbreak is located in zones {1a,1b,2a,2b}, for $\delta^{-1}=12$ h and $\beta_{0}=9\;m^{3}/\min$. Corresponding mean values *E*[*R*] are reported in Table IV. We note that zones 3*a* and 3*b* are *equivalent* to zones 1*a* and 1*b*, for all ventilation settings in Fig. 2, and thus we do not test them. For zones near the corridor (i.e., 1*b* and 2*b*), ventilation setting B is identified as the best one, while D is identified as the worst one. We note that ventilation setting B has no extract ventilation in zones 1*b* and 2*b* ($Q_{o,1b}=Q_{o,2b}=0$), but it directs the airflow instead toward corridor areas. In this setting B, corridor areas have no patients and significant ventilation, with airflow unbalance *from* bays *to* corridor areas acting in practice as an infection control measure. Thus, our results suggest that the spread control ability of a given ventilation setting depends on the location of the patient starting the outbreak as well as the airflow direction. However, from results in Fig. 4 and Table IV, ventilation setting D seems to perform poorly regardless of the initial infective location, suggesting that some ventilation settings might be inadvisable regardless of this location (i.e., if this location is unknown).

**Table IV:** Mean Values *E*[*R*] for Distributions in Fig. 4, That Is, for Different Locations of the Initial Infective

Location	A	B	C	D	E	F
1*a*	1.75	1.54	1.24	1.75	1.75	1.50
1*b*	1.74	1.28	1.57	1.93	1.50	1.73
2*a*	1.79	1.57	1.27	1.79	1.78	1.54
2*b*	1.78	1.32	1.62	1.98	1.54	1.78

When focusing on a particular location for the initial infective, comments above are supported by the sensitivity analysis on the ventilation parameters. For example, in Tables V and VI we report, for ventilation parameters $\theta \in \{\beta_{0},Q_{o,1a},\cdots ,Q_{o,c3},\beta_{Q1a,1b},\cdots ,\beta_{Qc3,3b}\}$, partial derivatives ∂E[R]/∂θ, and elasticities (∂E[R]/∂θ)·(θ/E[R]) for ventilation settings B and D, $\delta^{-1}=12$ h, $\beta_{0}=9\;m^{3}/\min$ and an infective patient starting the outbreak in zone 1*b*. We note that while dimensionless elasticities are useful for comparison purposes, they equal zero if parameter θ is zero.

**Table V:** Partial Derivatives and Elasticities of *E*[*R*] with Respect to Ventilation Parameters, for Setting B

Partial Derivatives						Elasticities					
β_0_	0.0230	$\beta_{Q2b,c2}$	−0.0141	$Q_{o,1b}$	−0.0890	β_0_	0.2899	$\beta_{Q2b,c2}$	−0.1042	$Q_{o,1b}$	0
$\beta_{Q1a,1b}$	−0.0201	$\beta_{Qc2,2b}$	0.0243	$Q_{o,c1}$	−0.0398	$\beta_{Q1a,1b}$	−0.1483	$\beta_{Qc2,2b}$	0	$Q_{o,c1}$	−0.2876
$\beta_{Q1b,1a}$	0.0207	$\beta_{Qc2,c3}$	0.0019	$Q_{o,2a}$	−0.0209	$\beta_{Q1b,1a}$	0	$\beta_{Qc2,c3}$	0	$Q_{o,2a}$	0
$\beta_{Q1b,c1}$	−0.0382	$\beta_{Qc3,c2}$	−0.0008	$Q_{o,2b}$	−0.0274	$\beta_{Q1b,c1}$	−0.2735	$\beta_{Qc3,c2}$	0	$Q_{o,2b}$	0
$\beta_{Qc1,1b}$	0.0301	$\beta_{Q3a,3b}$	−0.0032	$Q_{o,c2}$	−0.0221	$\beta_{Qc1,1b}$	0	$\beta_{Q3a,3b}$	−0.0240	$Q_{o,c2}$	−0.1615
$\beta_{Qc1,c2}$	0.0097	$\beta_{Q3b,3a}$	0.0060	$Q_{o,3a}$	−0.0120	$\beta_{Qc1,c2}$	0	$\beta_{Q3b,3a}$	0	$Q_{o,3a}$	0
$\beta_{Qc2,c1}$	−0.0041	$\beta_{Q3b,c3}$	−0.0081	$Q_{o,3b}$	−0.0159	$\beta_{Qc2,c1}$	0	$\beta_{Q3b,c3}$	−0.0601	$Q_{o,3b}$	0
$\beta_{Q2a,2b}$	−0.0056	$\beta_{Qc3,3b}$	0.0141	$Q_{o,c3}$	−0.0132	$\beta_{Q2a,2b}$	−0.0420	$\beta_{Qc3,3b}$	0	$Q_{o,c3}$	−0.0971
$\beta_{Q2b,2a}$	0.0106	$Q_{o,1a}$	−0.1089			$\beta_{Q2b,2a}$	0	$Q_{o,1a}$	0		

*Note*: Average individual detection time $\delta^{-1}=12$ h, global mixing rate $\beta_{0}=9\;m^{3}/\min$, and an initially infective patient starting the outbreak in zone 1*b*.

**Table VI:** Partial Derivatives and Elasticities of *E*[*R*] with Respect to Ventilation Parameters, for Setting D

Partial Derivatives						Elasticities					
β_0_	−0.0032	$\beta_{Q2b,c2}$	−0.0198	$Q_{o,1b}$	−0.1746	β_0_	−0.0151	$\beta_{Q2b,c2}$	0	$Q_{o,1b}$	0
$\beta_{Q1a,1b}$	0.0184	$\beta_{Qc2,2b}$	0.0241	$Q_{o,c1}$	−0.0872	$\beta_{Q1a,1b}$	0	$\beta_{Qc2,2b}$	0	$Q_{o,c1}$	−0.1356
$\beta_{Q1b,1a}$	−0.0248	$\beta_{Qc2,c3}$	0.0046	$Q_{o,2a}$	−0.0411	$\beta_{Q1b,1a}$	0	$\beta_{Qc2,c3}$	0	$Q_{o,2a}$	−0.1278
$\beta_{Q1b,c1}$	−0.0212	$\beta_{Qc3,c2}$	−0.0026	$Q_{o,2b}$	−0.0718	$\beta_{Q1b,c1}$	0	$\beta_{Qc3,c2}$	0	$Q_{o,2b}$	0
$\beta_{Qc1,1b}$	0.0124	$\beta_{Q3a,3b}$	0.0053	$Q_{o,c2}$	−0.0590	$\beta_{Qc1,1b}$	0	$\beta_{Q3a,3b}$	0	$Q_{o,c2}$	−0.0918
$\beta_{Qc1,c2}$	0.0167	$\beta_{Q3b,3a}$	−0.0074	$Q_{o,3a}$	−0.0293	$\beta_{Qc1,c2}$	0	$\beta_{Q3b,3a}$	0	$Q_{o,3a}$	−0.0912
$\beta_{Qc2,c1}$	−0.0090	$\beta_{Q3b,c3}$	−0.0140	$Q_{o,3b}$	−0.0516	$\beta_{Qc2,c1}$	0	$\beta_{Q3b,c3}$	0	$Q_{o,3b}$	0
$\beta_{Q2a,2b}$	0.0074	$\beta_{Qc3,3b}$	0.0171	$Q_{o,c3}$	0.0434	$\beta_{Q2a,2b}$	0	$\beta_{Qc3,3b}$	0	$Q_{o,c3}$	−0.0675
$\beta_{Q2b,2a}$	−0.0102	$Q_{o,1a}$	−0.1017			$\beta_{Q2b,2a}$	0	$Q_{o,1a}$	−0.3165		

*Note*: Average individual detection time $\delta^{-1}=12$ h, global mixing rate $\beta_{0}=9\;m^{3}/\min$, and an initially infective patient starting the outbreak in zone 1*b*.

Regime B requires airflow toward the corridor in order to expel pathogens from zone 1*b*, since $Q_{o,1b}=0$. Thus, rates $\beta_{Q1b,c1}$, $Q_{o,c1}$, $Q_{o,c2}$, and $Q_{o,c3}$ correspond to significantly large negative elasticities reported in Table V (i.e., increasing the values of these rates would lead to decreasing values of *E*[*R*]). Global mixing rate β_0_ has a significant impact (large positive elasticity) favoring disease spread, since increasing the value of β_0_ represents increasing the rate at which pathogens flow among all zones, instead of flowing specifically toward the extract ventilation areas (corridors in this setting).

According to results in Table VI, ventilation setting D could be significantly improved by increasing extract ventilation (especially in zones 1*a*, 1*b*, and *c*1), as well as increasing airflow from 1*b* to 1*a* and to *c*1. This is directly related to the fact that, since there is no extract ventilation in zone 1*b*, infectious material in this zone can only be expelled by directing it toward adjacent zones 1*a* and *c*1.

### 4.3. Decreasing Hospital Ward Infection Spread Risk Might Increase Risk at Specific Bays

It is clear that the number, *R*, of infections occurring until the end or detection of the outbreak can be split according to where these infections actually occur as

$$\begin{array}{ccc} R & = & R(1)+R(2)+R(3), \end{array}$$

where R(j) is the number of infections occurring at bay *j*. Although probabilities P(R(j)=n) can be analytically computed by adapting arguments in Section 3, details are omitted here for the sake of brevity, and results in Table VII are obtained from 10^6^ stochastic simulations of the process.

**Table VII:** Mean Values of *E*[*R*], E[R(1)], E[R(2)], and E[R(3)] for $\beta_{0}=9\;m^{3}/\min$, $\delta^{-1}=48$ h and Ventilation Settings A, D, and E; Initially Infective Individual in Zone 1*a*

Regime	*E*[*R*]	E[R(1)]	E[R(2)]	E[R(3)]
A	5.01	2.65	1.34	1.02
D	5.20	2.56	1.47	1.17
E	4.86	2.69	1.25	0.92

In Table VII, we report values of E[R]=E[R(1)]+E[R(2)]+E[R(3)] for $\beta_{0}=9\;m^{3}/\min$, $\delta^{-1}=48$ h, ventilation settings A, D, and E, and an infective patient starting the outbreak in zone 1*a*. Results suggest that epidemic spread can be limited by *switching* ward ventilation from setting D to A, and further containment is obtained by switching to ventilation setting E. However, infection risk in bay 1 (in terms of E[R(1)]) behaves contrarily; although the global hospital ward infection risk (in terms of *E*[*R*]) is lower for setting E, this is at the expense of expelling pathogens from the infected zone 1*a* toward zones 1*b* and *c*1, and thus posing a greater risk to patients in bay 1.

### 4.4. Interplay with Detection Management

**Figure 5:**
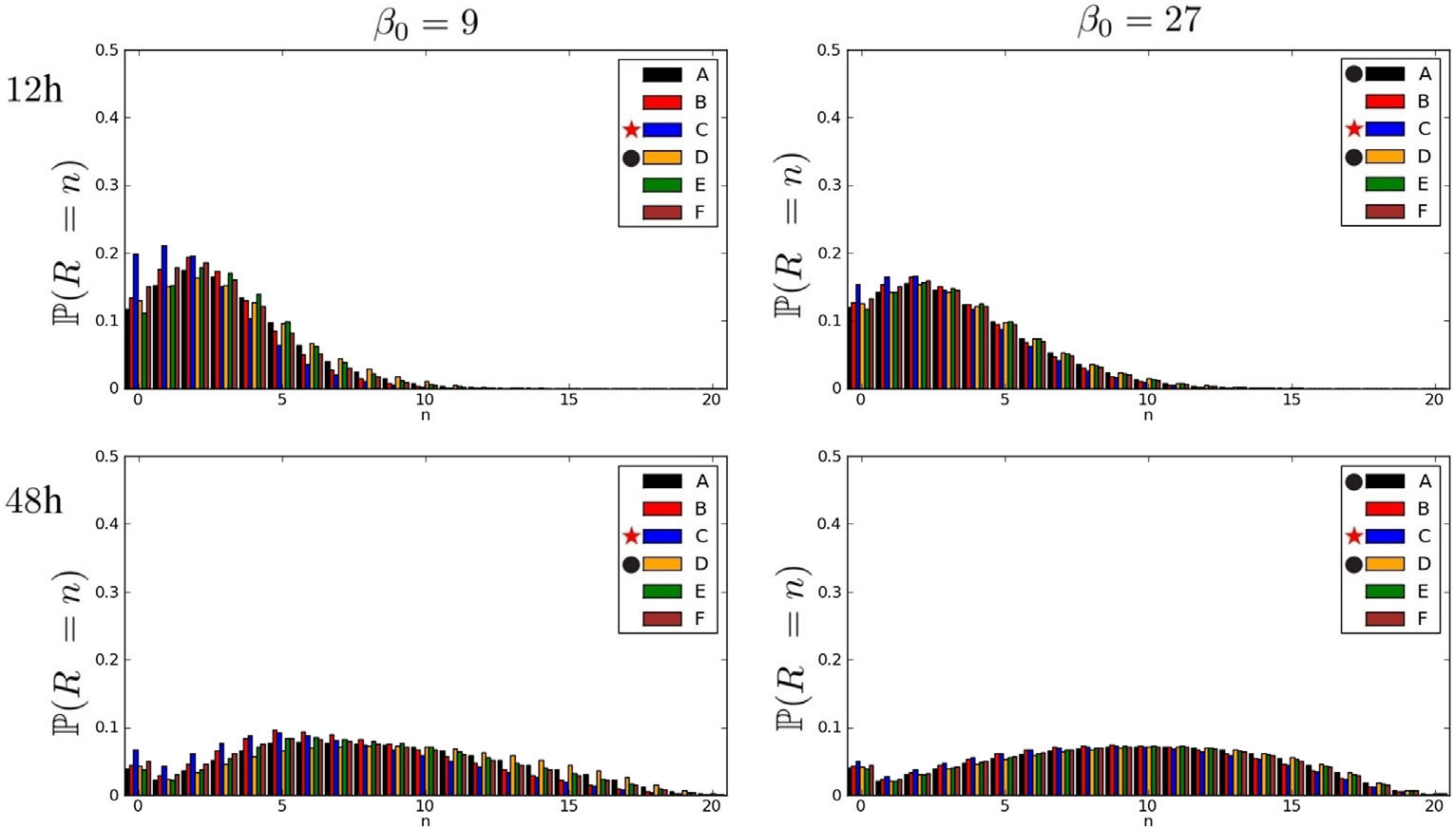
Probability mass function of the number *R* of infections until the end or detection of the outbreak, for ventilation settings A–F. Declaration of the outbreak occurs once two patients show symptoms, and each infective patient shows symptoms after an average time $\delta^{-1}\in \left\{12\;h,48\;h\right\}$. Global mixing rate $\beta_{0}\in \left\{9,27\right\}\;m^{3}/\min$. Initial infective located in zone 1*a*.

As explained in Section 3.3, detection and declaration of an outbreak in the hospital ward may require several patients showing symptoms, and not only one. In Fig. 5, we plot analogous results to those in Fig. 3 when declaration of the outbreak occurs after D=2 patients show symptoms (each after average time $\delta^{-1}$). The corresponding mean values of *E*[*R*] are reported in Table VIII.

**Table VIII:** Mean Values of *E*[*R*] for Scenarios in Fig. 5

β_0_	$\delta^{-1}$	A	B	C	D	E	F
9	12 h	3.20	2.78	2.33	3.26	3.14	2.79
	48 h	8.42	7.31	6.78	8.79	8.10	7.72
27	12 h	3.52	3.28	3.07	3.50	3.49	3.34
	48 h	9.37	8.87	8.63	9.39	9.26	9.10

We note that values in Table VIII are significantly larger than those in Table III, since outbreak declaration takes longer to occur, allowing for more infections to take place. This increase is significantly larger than the differences that can be observed, in Table III, between different ventilation settings, suggesting again that detection policy is likely to dominate ventilation as an infection control strategy. Under slow detection scenarios ($\delta^{-1}=48$ h), we observe in Fig. 5 a clear bimodality for the distribution of *R*. Thus, our model predicts that under slow detection, a two‐output situation can be expected: either the initially infective patient is discharged before infecting any other patient (so that R=0), or this patient infects a second patient, leading to a large outbreak (represented by the second mode in Fig. 5).

### 4.5. Screening at Admission

In Sections 4.1–4.4, we analyze infection spread under the assumption that each individual in zone *j* is detected (by showing symptoms) at rate $\delta_{j}$, with $\delta_{j}=\delta$ for all *j*, and where the outbreak is detected and declared after one (or several) infective patients are detected. This leads to the contribution $\sum_{j=1}^{M}\delta_{j}i_{j}$ in Equations (4) and (5). However, if the detection of the outbreak is due instead to the screening of the newly admitted patient who starts the outbreak, and results of this screening arrive after an average time $\delta^{-1}$, then the outbreak is detected at rate δ, and one needs to replace $\sum_{j=1}^{M}\delta_{j}i_{j}$ by δ in Equations (4) and (5).

**Figure 6:**
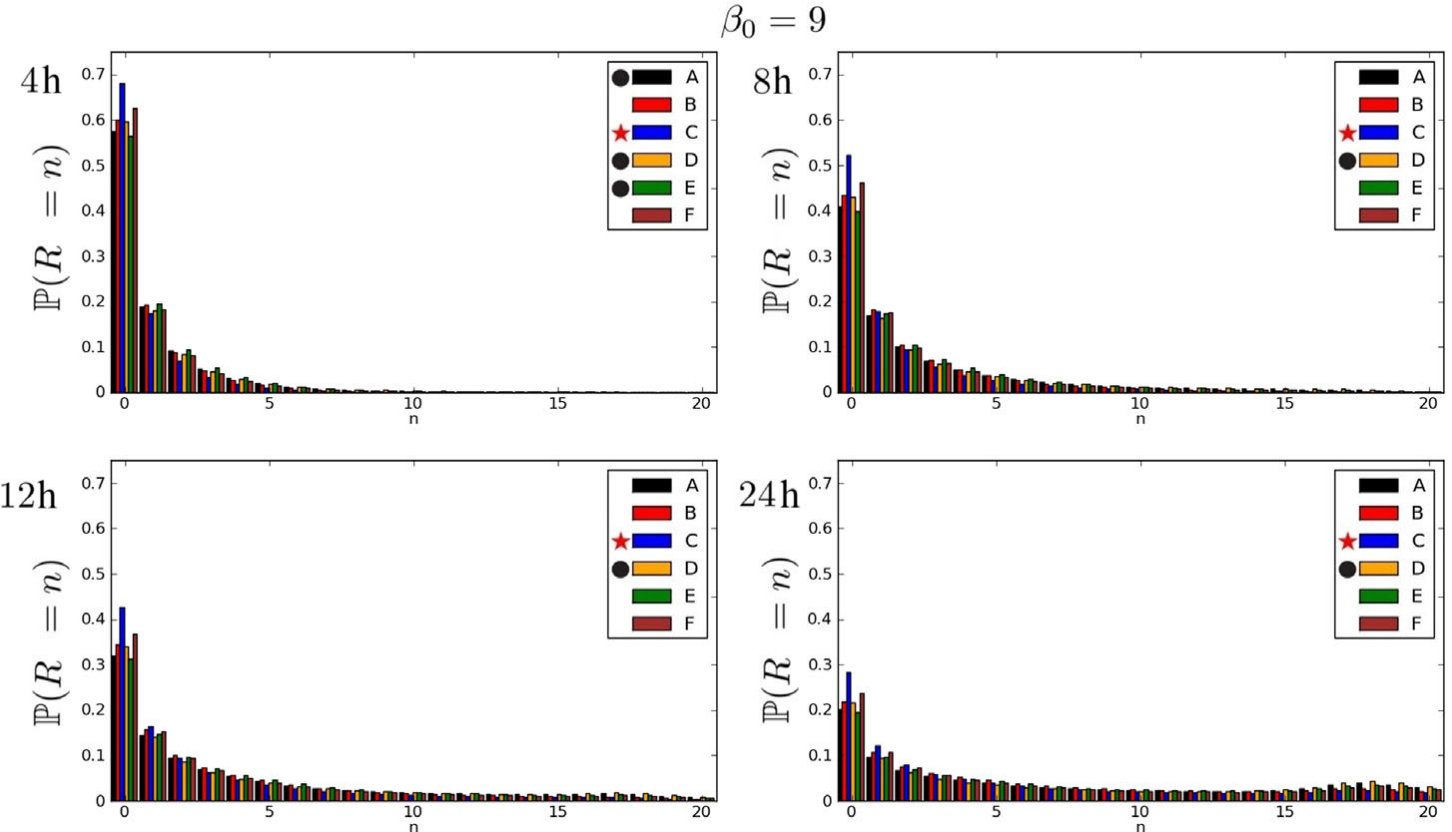
Probability mass function of the number *R* of infections until the end or detection of the outbreak, for ventilation settings A–F. Declaration of the outbreak occurs after an average time $\delta^{-1}\in \left\{4\;h,8\;h,12\;h,24\;h\right\}$, which is independent of the number of infectives and represents a screening event. Global mixing rate $\beta_{0}=9\;m^{3}/\min$. Initial infective located in zone 1*a*.

Under this hypothesis, we plot in Fig. 6 the probability mass function of the number *R* of infections until the end or detection of the outbreak, when the results of this screening (and thus, the declaration of the outbreak) arrive after an average time $\delta^{-1}\in \left\{4\;h,8\;h,12\;h,24\;h\right\}$. Corresponding mean values of *E*[*R*] are reported in Table IX. If results arrive after $\delta^{-1}=4$ h, ventilation has a less significant impact on the nosocomial spread, and low values of *E*[*R*] are reported in Table IX. The number of infections until the end or detection of the outbreak proportionally increases with the delay $\delta^{-1}$ in obtaining the screening results. In particular, for $\delta^{-1}=24$ h significant differences in *E*[*R*] can be noticed among the different ventilation settings, and a marked bimodality can be observed in Fig. 6.

**Table IX:** Mean Values *E*[*R*] for Scenarios in Fig. 6

$\delta^{-1}$	A	B	C	D	E	F
4 h	1.12	0.92	0.66	1.11	1.11	0.89
8 h	2.66	2.13	1.63	2.72	2.59	2.16
12 h	4.24	3.43	2.74	4.36	4.11	3.54
24 h	8.15	6.91	5.94	8.36	7.91	7.17

### 4.6. Ventilation and Overoccupancy

In this subsection, our aim is to shed some light on the interplay between ventilation, nosocomial spread, and overoccupancy of the hospital ward. We represent hospital ward overoccupancy by locating three additional patients at the corridor areas; in particular, we set N=21 and locate one additional patient in each of the {c1,c2,c3} zones. This practice is common in U.K. hospitals during times of high demand. We assume that the outbreak is detected and declared after the first patient shows symptoms, each patient showing symptoms after an average time $\delta^{-1}=12$ h. For interzonal mixing $\beta_{0}=9\;m^{3}/\min$, we report in Table X the mean number *R* of infections until the end or detection of the outbreak, for ventilation settings A–F, and for the initial infective patient being located in zones {1a,c1,c2,c3}.

**Table X:** Mean Values *E*[*R*] for a Hospital Ward with Overoccupancy

Location of Initial Infective	A	B	C	D	E	F
1*a*	2.01	1.82	1.37	2.04	2.02	1.72
*c*1	1.95	1.14	1.95	2.19	1.71	1.92
*c*2	1.99	1.18	2.00	2.23	1.75	1.96
*c*3	1.95	1.14	1.95	2.19	1.71	1.92

*Note*: Patients show symptoms after an average time $\delta^{-1}=12$ h, and we set $\beta_{0}=9\;m^{3}/\min$.

We first note that results in Table X are exactly the same for an initially infective individual being located in zones *c*1 and *c*3. This is explained by noting that bays 1 and 3 are completely symmetric for all ventilation settings as noted in Section 4.2; see diagrams and matrices in Fig. 2. On the other hand, when the initial infective patient is located in zone 1*a*, results in Table X can be compared to those in Table III for $\left(\delta^{-1},\beta_{0}\right)=\left(12\;h,9\;m^{3}/\min\right)$. For zone 1*a*, values of *E*[*R*] are larger in Table X than in Table III; if the nosocomial outbreak is initiated by an infective patient in zone 1*a*, more infections during this outbreak should be expected under ward overoccupancy. This might not be only related to having more patients in the ward under overoccupancy (21 instead of 18), but also to the potential of patients in the corridor to act as *infection links* between bays. For example, under overoccupancy, an infective individual in zone 1*a* might infect individuals in bay 2 by, as a first step, infecting individuals in the corridor areas. These people then might more easily infect individuals in bay 2, before being discharged, due to being in closer proximity and depending on the particular ventilation setting in place in the ward.

Infection dynamics related to the scenario above highly depend on the particular ventilation setting under study, which can be noticed by inspecting rows corresponding to zones {c1,c2,c3} in Table X. While an individual in zone 1*a* has a larger infectious potential (in terms of *E*[*R*]) than individuals located in the corridor when ventilation settings B and E are in place, this is not the case for ventilation settings C, D, and F, and these infectious potentials are comparable under ventilation setting A, which represents a well‐mixed scenario. Our results then indicate that overoccupancy leads in general to higher airborne spread risks, and that this increase can be especially significant depending on the specific ventilation in place.

### 4.7. The Unit of Infection

We note that parameter *q* is highly pathogen dependent, ranging from q∼0.01 quanta/min for rhinovirus to q∼10 quanta/min for measles (Noakes & Sleigh, 2009). We perform a parametric analysis by varying *q* to assess the sensitivity of our conclusions, and report expected infections *E*[*R*] until the end of the outbreak in Table XI. In particular, we are interested in the mean number *E*[*R*] of infections if the outbreak is detected on the first patient showing symptoms, each patient showing symptoms after an average time $\delta^{-1}=12$ h, and where we consider $\beta_{0}=9$ m^3^/min. We note that, as expected, increasing values of *q* lead to increasing mean number *E*[*R*] of infections. However, this does not seem to affect the relative infectiousness of ventilation setting C, which is identified as the best scenario regardless of the value of *q*. On the other hand, less advantageous ventilation schemes are dependent on the value of *q*. For example, ventilation setting A and E can be identified as the worst for q=0.1 quanta/min, while setting D can be seen as the worst one for q=50.0 quanta/min.

**Table XI:** Mean Number *E*[*R*] of Infections until End or Declaration of the Outbreak, for $\beta_{0}=9$ m^3^/min and When Outbreak Declaration Occurs After the First Patient Shows Symptoms

*q*	A	B	C	D	E	F
0.1	0.41	0.36	0.27	0.38	0.42	0.34
0.5	1.75	1.54	1.24	1.75	1.74	1.50
1.0	3.06	2.66	2.26	3.14	3.00	2.69
10.0	11.43	10.44	10.16	11.75	11.13	10.92
50.0	15.54	15.12	15.05	15.66	15.41	15.35

*Note*: Each patient shows symptoms after an average time $\delta^{-1}=12$ h. Different values of *q* (quanta/min) considered.

## 5. DISCUSSION AND CONCLUSIONS

In this work, we link a zonal ventilation model for the generation and airborne spread of infectious material within a hospital ward with a multicompartment SIS Markovian model for the infection of patients within this ward. Our model incorporates the possibility of considering a wide range of ventilation settings, the discharge and arrival of patients within the ward, as well as different hypotheses regarding how outbreak detection and declaration occurs. Moreover, it allows us to explore the interplay between ventilation, outbreak management, ward overoccupancy, and the location of the infective patient starting the outbreak.

Our results suggest that detection time dominates ventilation when the variable of interest is the number of infections occurring before the declaration or end of the outbreak, with longer detection times leading to significantly more infections happening. Longer detection times can arise when analyzing pathogens with long infectious asymptomatic periods, when declaration of an outbreak requires for several patients to show symptoms, or when this declaration depends on screening events for which results take longer to arrive. The interplay between ventilation of the hospital ward and location of the initially infective patient starting the outbreak implies that recommendations on where to locate potentially infected (e.g., newly admitted) patients in a given hospital ward could be issued depending on the ventilation in place in the ward. Our model also predicts that decreasing the infection spread risk in the hospital ward can sometimes come at the expense of increasing the risk in particular areas of the ward.

Similar models have already been considered in the literature for linking zonal ventilation scenarios with epidemic spread models (Carruthers et al., 2018; Ko et al., 2004; Noakes & Sleigh, 2009), where epidemic dynamics are usually analyzed by means of stochastic simulations. To the best of our knowledge, this is the first time that this link is carried out by defining in detail the CTMC for the infection spread, where infection rates at each ventilation zone are in fact functions $\lambda_{j}\left(i_{1},\cdots ,i_{M}\right)$ of the number of infectives in each zone at any given time, and where $\left(i_{1},\cdots ,i_{M}\right)$ represents in fact a state of the CTMC under study. This detailed mathematical construction allows for the analytical computation of summary statistics (such as *R* in this work), and for carrying out a local sensitivity analysis that allows one to identify the particular factors of each ventilation setting having the most significant impact on the infection spread.

It should be noted that the primary objective in this study is to demonstrate this detailed mathematical analysis and how it can be applied to evaluate the relative influence of different parameters. The model is applied to a hypothetical hospital ward, which, while it is representative of multibed ward environments in many hospitals, is a very simplified model of reality. The results demonstrate that the ventilation flow settings may influence the dispersion of airborne pathogens and hence the risk of transmission; however, these should be interpreted with caution. We assume a steady‐state ventilation scenario with the flow pattern replicated exactly between neighboring bays 1, 2, and 3. In reality, the flows will not be exactly identical for every bay, and other factors such as heat sources and movement of people will alter the mixing with and between zones. In particular, corridor ventilation often has a directional flow due to wider spacing of ventilation supply/extract grilles, which may hinder or improve the control of infection. However, the analysis we have carried out gives some clear insight into why particular directional flows influence risk, and the relative importance of detection strategies, ventilation control, and occupancy.

It is clear that some of our conclusions could be highly dependent on the hospital ward structure, and therefore the flexibility of our methodology comes into play. It can be applied to any hospital ward of interest by appropriately adapting the corresponding ventilation matrix **V**. Although carrying out a detailed mathematical analysis of a number of potentially different hospital ward structures is out of the scope of this article, we include a short numerical study of an alternative hospital ward in the Supplementary Material. The aim of this is twofold: (i) to show how our methodology can be easily implemented for a different hospital ward to that in Fig. 2 by just adapting the ventilation matrix **V**, illustrating how this matrix varies with hospital ward structure; and (ii) to show that while some of our conclusions might be hospital ward structure dependent, others seem to be valid for a wide range of hospital ward structures (e.g., detection dominates ventilation as well in this alternative hospital ward).

In this article, we go beyond the SI epidemic model in Noakes and Sleigh (2009), proposing an SIS‐type model, which allows us to incorporate patients' arrival/discharge and outbreak detection and declaration. This is similar to the model recently proposed by López‐García and Kypraios (2018), as a unified framework for modeling the spread of nosocomial infections. We note that this epidemic model structure would be especially relevant for pathogens with no or short (i.e., negligible compared to the average patient's LOS) noninfectious or latent periods, and where the infectious period is long enough so that recovery of patients does not occur before discharge (or detection). Depending on the hospital ward under analysis and the average LOS of patients in this ward, this could be the case for influenza or norovirus. Pathogens with nonnegligible incubation periods (e.g., 7–21 days for measles) might require more complex stochastic epidemic model structures such as the SEIR (*susceptible–exposed–infective–recovered*). On the other hand, when analyzing hospital wards with longer average patient LOS, so that individuals may become infected and recover during their stay, SIRS‐type epidemic models would be required to represent the recovery of patients (I→R) before discharge (R→S) occurs. We note here that in principle, the methodology outlined in Section 3 can be extended to any of these compartmental‐based epidemic models for the disease spread dynamics, where the link between the deterministic zonal ventilation model for the airflow dynamics and the stochastic epidemic model for disease spread dynamics would still be as in Section 2. In a similar way, more complex epidemic model structures could allow one to study the infection spread dynamics after outbreak detection and declaration occurs. In this article, we have focused instead on the impact of ventilation on disease spread until the end or declaration of the outbreak. Considering these alternative compartmental‐based epidemic model structures could be the aim of future work.

Finally, we note that when carrying out our analysis, the main computational effort lies in solving systems of linear equations, where the number of equations is determined by the number of states of the corresponding CTMC, given by Equation (6) in our model. Limitations of our approach are then of a computational nature, since highly complex epidemic models (here, a multicompartment SIS stochastic model with detection) linked to large hospital wards split in many different ventilation zones (here, M=9 zones with three *empty* zones and six zones containing three patients each) would lead to an intractable number of equations, and stochastic simulation approaches would prevail.

## 6. DATA, SOFTWARE, AND REPRODUCIBILITY

Computer codes (in *Python*) in order to reproduce our numerical results are available López‐García, King, and Noakes (2019).

## Supporting information


**Fig. S1**. Diagrams of the ventilation settings considered, and corresponding ventilation matrices V.
**Fig. S2**. Probability mass function of the number *R* of infections until the end or detection of the outbreak, for ventilation settings *SA*‐*SD*.
**Table S1**. Mean Values of *E*[*R*] for Scenarios in Fig. S2

## Footnotes

1
#C represents the number of elements (states) in C.

## APPENDIX 1

### A.1. First‐Step Arguments

We explain here in detail how Equation (4) is obtained, in order to compute probabilities

$$\begin{array}{ccc} & & p_{\left(i_{1},i_{2},\cdots ,i_{M}\right)}\left(n\right)=P\left(R=n\;|\;\left(I_{1}\left(0\right),I_{2}\left(0\right),\cdots ,I_{M}\left(0\right)\right)\right)\\ & & \;\;\;\;\;\;=(\left(i_{1},i_{2},\cdots ,i_{M}\right)) \end{array}$$

for any initial state $(i_{1},\cdots ,i_{M})\in C$. These equations are obtained by following a first‐step argument, and applying the total probability law regarding the following event occurring in the stochastic process. In particular, let us denote by $\left(i_{1},i_{2},\cdots ,i_{j},\cdots ,i_{M}\right)\to \left(i_{1},i_{2},\cdots ,i_{j}+1,\cdots ,i_{M}\right)$ the event representing that, if the process is initially at state $\left(i_{1},i_{2},\cdots ,i_{j},\cdots ,i_{M}\right)$, it moves to state $\left(i_{1},i_{2},\cdots ,i_{j}+1,\cdots ,i_{M}\right)$ in the next *jump* (i.e., that we have $\left(i_{1},i_{2},\cdots ,i_{j},\cdots ,i_{M}\right)$ infectives within the hospital ward at current time, and the next event that occurs is an infection in zone *j*). From the theory of CTMCs, it is well known that the probability of this event occurring is:

$$\begin{array}{ccc} & & P(\left(i_{1},i_{2},\cdots ,i_{j},\cdots ,i_{M}\right)\to \left(i_{1},i_{2},\cdots ,i_{j}+1,\cdots ,i_{M}\right))\\ & & \;\;\;=\frac{\lambda_{j}\left(i_{1},\cdots ,i_{M}\right)\left(N_{j}-i_{j}\right)}{\sum_{k}\left(\lambda_{k}\left(i_{1},\cdots ,i_{M}\right)\left(N_{k}-i_{k}\right)+\left(\gamma_{k}+\delta_{k}\right)i_{k}\right)}; \end{array}$$

that is, it is equal to the ratio between the rate corresponding to this event and the sum of all the rates corresponding to all the possible events that can actually occur (infection in any zone, discharge in any zone, or detection of an infective individual in any zone). The same argument applies to any other possible event, so that $P(\left(i_{1},i_{2},\cdots ,i_{j},\cdots ,i_{M}\right)\to \left(i_{1},i_{2},\cdots ,i_{j}-1,\cdots ,i_{M}\right))$ is given by

$$\frac{\gamma_{j}i_{j}}{\sum_{k}\left(\lambda_{k}\left(i_{1},\cdots ,i_{M}\right)\left(N_{k}-i_{k}\right)+\left(\gamma_{k}+\delta_{k}\right)i_{k}\right)}$$

and $P(\left(i_{1},i_{2},\cdots ,i_{j},\cdots ,i_{M}\right)\to \Delta )$ is given by

$$\frac{\sum_{j}\delta_{j}i_{j}}{\sum_{k}\left(\lambda_{k}\left(i_{1},\cdots ,i_{M}\right)\left(N_{k}-i_{k}\right)+\left(\gamma_{k}+\delta_{k}\right)i_{k}\right)}.$$

Thus, we can apply the total probability law so that $p_{\left(i_{1},\cdots ,i_{M}\right)}\left(0\right)=P\left(R=0\right)$ is equal to

$$\begin{array}{ccc} & & \sum_{k=1}^{M}(P\left(R=0\;|\;\left(i_{1},\cdots ,i_{M}\right)\to \left(i_{1},\cdots ,i_{k}+1,\cdots ,i_{M}\right)\right))\\ & & P(\left(i_{1},\cdots ,i_{M}\right)\to \left(i_{1},\cdots ,i_{k}+1,\cdots ,i_{M}\right))\\ & & +\;(P\left(R=0\;|\;\left(i_{1},\cdots ,i_{M}\right)\to \left(i_{1},\cdots ,i_{k}-1,\cdots ,i_{M}\right)\right))\\ & & \left(P\left(\left(i_{1},\cdots ,i_{M}\right)\to \left(i_{1},\cdots ,i_{k}-1,\cdots ,i_{M}\right)\right)\right)\\ & & +\;P\left(R=0\;|\;\left(i_{1},\cdots ,i_{M}\right)\to \Delta \right)P\left(\left(i_{1},\cdots ,i_{M}\right)\to \Delta \right)\\ & & =\frac{1}{\sum_{j=1}^{M}\left(\lambda_{j}\left(i_{1},\cdots ,i_{M}\right)\left(N_{j}-i_{j}\right)+\left(\gamma_{j}+\delta_{j}\right)i_{j}\right)}\\ & & \sum_{k=1}^{M}\left(\gamma_{k}i_{k}p_{\left(i_{1},\cdots ,i_{k}-1,\cdots ,i_{M}\right)}\left(0\right)+\delta_{k}i_{k}\right), \end{array}$$

corresponding to Equation (4).

### A.2. Algorithms

Algorithm For computing the probability distribution of *R*
For all $(i_{1},\cdots ,i_{M})\in C$, compute and store $\lambda_{j}\left(i_{1},\cdots ,i_{M}\right)$, for 1≤j≤M, from Equation (3);
n=0;
$p_{\left(0,0,\cdots ,0\right)}\left(0\right)=1$; for I=1,2,…,N: for all $i=(i_{1},i_{2},\cdots ,i_{M})$ s.t. $\sum_{j=1}^{M}i_{j}=I$:           $p_{i}\left(0\right)=\frac{1}{\sum_{j}\left(\lambda_{j}\left(i_{1},\cdots ,i_{M}\right)\left(N_{j}-i_{j}\right)+\left(\gamma_{j}+\delta_{j}\right)i_{j}\right)}$
                $\times \sum_{k}\left(\gamma_{k}i_{k}p_{\left(i_{1},\cdots ,i_{k}-1,\cdots ,i_{M}\right)}\left(0\right)+\delta_{k}i_{k}\right)$
for n=1,2,3,⋯:   $p_{\left(0,0,\cdots ,0\right)}\left(n\right)=0$;   for I=1,2,⋯,N:      for all $i=(i_{1},i_{2},\cdots ,i_{M})$ s.t. $\sum_{j=1}^{M}i_{j}=I$:         $p_{i}\left(n\right)=\frac{1}{\sum_{j}\left(\lambda_{j}\left(i_{1},\cdots ,i_{M}\right)\left(N_{j}-i_{j}\right)+\left(\gamma_{j}+\delta_{j}\right)i_{j}\right)}$
            $\times \sum_{k}(\gamma_{k}i_{k}p_{\left(i_{1},\cdots ,i_{k}-1,\cdots ,i_{M}\right)}\left(n\right)+\lambda_{k}\left(i_{1},\cdots ,i_{M}\right)$
            $\times \;\left(N_{k}-i_{k}\right)p_{\left(i_{1},\cdots ,i_{k}+1,\cdots ,i_{M}\right)}\left(n-1\right))$.
