# Supplementary material for: A Multicompartment SIS Stochastic Model with Zonal Ventilation for the Spread of Nosocomial Infections: Detection, Outbreak Management, and Infection Control
Source: Risk Anal. 2019 Mar 29;39(8):1825–42. doi: 10.1111/risa.13300 (PMC6850612; doi:10.1111/risa.13300)
Supplement: Supplementary file 1 — Fig. S1. Diagrams of the ventilation settings considered, and corresponding ventilation matrices V. Fig. S2. Probability mass function of the number R of infections until the end or detection of the outbreak, for ventilation settings SA‐SD. Table S1. Mean Values of E[R] for Scenarios in Fig. S2 [file RISA-39-1825-s001.pdf]

A multicompartment SIS stochastic model with zonal ventilation for the spread of nosocomial infections: detection, outbreak management and infection control

Martín López-García[1]*, Marco-Felipe King[2], Catherine J. Noakes[2]

[1]Department of Applied Mathematics, School of Mathematics, University of Leeds, LS2 9JT Leeds, UK

[2]Institute for Public Health and Environmental Engineering, School of Civil Engineering, University of Leeds, LS2 9JT Leeds, UK

Supplementary Material

Abstract

In this supplementary material we show how our methodology can be implemented for a different hospital ward to that in [1, Figure 2]. We consider a hospital ward with six rooms with patients allocated across these rooms in an asymmetrical way. In particular, rooms 1, 3 and 5 contain one patient each, while rooms 2, 4 and 6 contain three patients each. We consider a number of ventilation settings (SA, SB, SC and SD), and carry out a number of numerical simulations to explore how airflow dynamics interplay with other factors related to infection spread, such as outbreak detection. The main aim is: (i) to show how our methodology can easily be extended to any hospital ward structure by appropriately modifying the ventilation matrix $\mathbf{V}$; and (ii) to show how, while some of our conclusions in [1] seem to be valid also for the hospital ward structure considered in this supplementary material, others might highly depend on the particular ward structure under study.

1 An alternative hospital ward structure

We consider the hospital ward outlined in Figure S1 with four potential ventilation settings. We consider that rooms 1, 3 and 5 have one patient each, while rooms 2, 4 and 6 have three patients each, so that the total number of patients is $N = 12$, since no patients are allocated in corridor areas. We analyse the situation where an infected individual in room 1 starts the nosocomial outbreak and set, for illustrative purposes, parameter values $p_i = 0.01\ m^3 \cdot min^{-1}$, $q_i = 0.5\ quanta \cdot min^{-1}$ and $\gamma_i^{-1} = 7\ days$ for all patients in all zones as in [1, Section 4]. Ventilation rate over the whole ward is, as in [1], $27\ m^3 \cdot min^{-1}$ which equates to an air change rate of $3\ AC \cdot h^{-1}$. Our aim is to show how our methodology can be easily adapted

*Corresponding author: m.lopezgarcia@leeds.ac.uk

for any hospital ward by appropriately modifying the corresponding ventilation matrix $\mathbf{V}$. For $\beta_0 = 9\ m^3 \cdot min^{-1}$, matrix $\mathbf{V}$ is reported in Figure S1 for each ventilation setting considered in this supplementary material. We note that, in order to construct this matrix, rooms (i.e. rows and columns of this matrix) are ordered as

$$1 \prec 2 \prec c1 \prec 3 \prec 4 \prec c2 \prec 5 \prec 6 \prec c3.$$

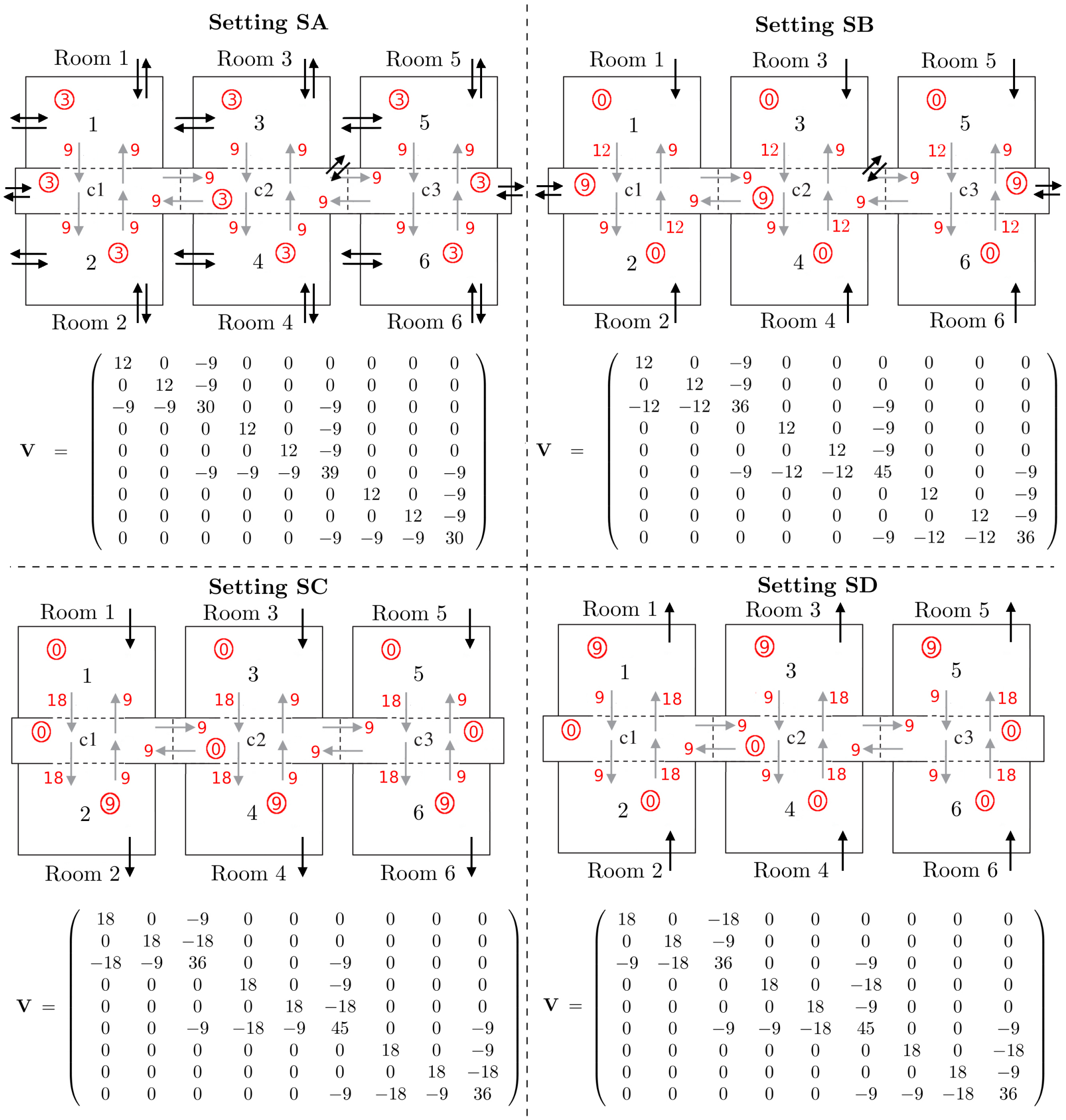

Figure S1: Diagrams of the ventilation settings considered, and corresponding ventilation matrices $\mathbf{V}$ (for $\beta_0 = 9\ m^3 \cdot min^{-1}$). Grey arrows: ventilation between zones (β_{ij} -$m^3 \cdot min^{-1}$-); Black arrows: ventilation supply and extract to the ward; Circled values: extract ventilation rates ($Q_{o,j}$ -$m^3 \cdot min^{-1}$-)

We note here that although all the detailed analysis carried out in [1, Section 4] for the hospital ward in [1, Figure 2] can also be carried out for our hospital ward in Figure S1, we focus in this supplementary material, for the sake of brevity, on the interplay between airflow dynamics and outbreak detection policies. For this, we implement here the analogous analysis to that in [1, Subsection 4.4], to the hospital ward and ventilation settings represented in Figure S1. In particular, under the assumption that the detection and declaration of the outbreak

occurs after two patients showing symptoms (each patient showing symptoms after an average time δ^{-1}), we plot in Figure S2 (analogous to [1, Figure 5]) the probability mass functions of the number R of infections until outbreak declaration, for ventilation settings $SA - SD$. Mean values of these distributions are reported in Table S1 (analogous to [1, Table VIII]).

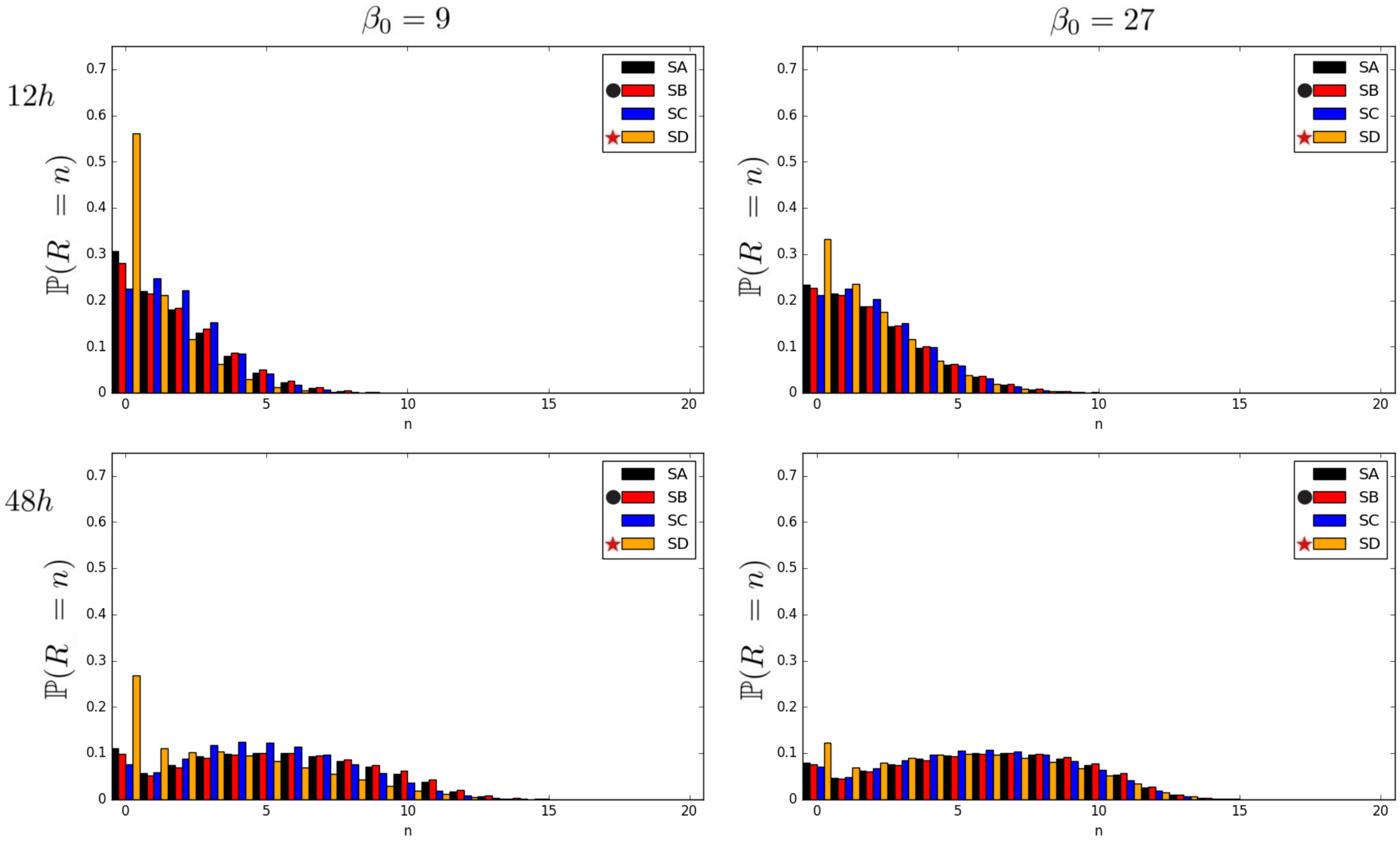

Figure S2: Probability mass function of the number R of infections until the end or detection of the outbreak, for ventilation settings $SA - SD$. Declaration of the outbreak occurs once two patients show symptoms, and each infective patient shows symptoms after an average time $\delta^{-1} \in \{12h, 48h\}$. Global mixing rate $\beta_0 \in \{9, 27\}$ $m^3 \cdot min^{-1}$. Initial infective located at room 1

Table S1: Mean values of $E[R]$ for scenarios in Figure S2.

β_0	δ^{-1}	SA	SB	SC	SD
9	12h	1.77	1.90	1.87	0.86
	48h	5.24	5.49	4.91	3.26
27	12h	2.13	2.19	2.12	1.62
	48h	5.92	6.03	5.71	5.04

In general, ventilation setting SD is identified as the best for controlling infection spread, while ventilation settings SA, SB and SC can be identified as the least effective for this purpose. This is directly related to the location of the initial infective (room 1), the airflow dynamics represented by these settings, and the (asymmetric) location of patients within the ward. It is to be expected that setting SD maintains concentration of pathogen in the air low for rooms 2, 4 and 6, which are the ones containing three patients each, leading to small outbreaks.

It does this by directing the air from these overpopulated rooms toward rooms 1, 3 and 5, which contain only one patient each. On the other hand, ventilation settings leading to larger pathogen concentrations in rooms 2, 4 and 6 (settings SA, SB and SC) lead to larger outbreaks.

We should note here that, as explained above, some differences in Figure S2 and Table S1 between different ventilation settings can be explained due to asymmetries in this hospital ward structure (e.g. where not all the rooms are equally important having the same amount of patients each), while the ward in [1] was symmetric. There is a clear interplay, as observed as well in [1, Figure 5], between the ventilation setting and the outbreak detection policy, where ventilation settings SA and SC behave better or worse depending on how quickly outbreak detection occurs. Once again, detection seems to be more important than ventilation from an infection control perspective according to results in Figure S2 and Table S1.

Our results and comments above suggest that, while some of our conclusions in [1] might be valid in a general sense (i.e. for wide ranges of hospital ward structures), others might be highly dependent on hospital ward structure. The flexibility of our methodology, which can be implemented to any hospital ward by just adjusting the ventilation matrix $\mathbf{V}$ accordingly, can then allow one to carry out a detailed analysis of the infection spread dynamics for each particular hospital ward, and to study the potential impact on these dynamics of outbreak management, ventilation settings, detection (e.g. surveillance) policies and patients allocation.

References

[1] López-García M, King M-F, Noakes CJ (2019) A multicompartment SIS stochastic model with zonal ventilation for the spread of nosocomial infections: detection, outbreak management and infection control. Risk Analysis, DOI: 10.1111/risa.13300
